# How Match-Related Variables Influence the Physical Demands of Professional Female Soccer Players during the Regular Season

**DOI:** 10.3390/jfmk9030149

**Published:** 2024-08-28

**Authors:** Lorenzo Marcelli, Fioretta Silvestri, Gianluca Di Pinto, Maria Chiara Gallotta, Davide Curzi

**Affiliations:** 1 Department of Humanities, Movement and Education Sciences, University “Niccolò Cusano”, 00166 Rome, Italy; lorenzo.marcelli@unicusano.it (L.M.); fioretta.silvestri@unicusano.it (F.S.); gianluca.dipinto@phd.unich.it (G.D.P.); davide.curzi@unicusano.it (D.C.); 2 BIND—Behavioral Imaging and Neural Dynamics Center, University “G. D’Annunzio” Chieti-Pescara, 66100 Chieti, Italy; 3 Department of Medicine and Aging Sciences, University “G. D’Annunzio” Chieti-Pescara, 66100 Chieti, Italy; 4 Department of Physiology and Pharmacology “Vittorio Erspamer”, Sapienza University of Rome, 00185 Rome, Italy

**Keywords:** soccer, external workload, female, match-related factor, professional, player role, yo-yo IR1, GPS, HR, CMJ

## Abstract

To investigate how contextual or environmental factors may influence the athletic performance of female soccer players during competitions, this study aimed to assess the impact of match-related variables (playing surface; opponent levels; opponent result trends; match status, and match outcomes) on the athletic performance of professional female soccer athletes. Seventeen athletes (25.5 ± 4.3 years of age) from the same team competing in the Italian second division were divided into two groups according to their roles and athletic characteristics: Group A (central defenders and forwards) and Group B (right/left full-backs and midfielders). Total distance (TD) and sprint distance (SD); high-speed running (HSR), acceleration (ACC), and deceleration numbers (DEC); average and maximal heart rate (HR_avg; HR_max); and match-related factors were collected during the 22 regular season matches. A *T*-test and ANOVA were used to calculate the differences between groups in GPS and HR variables and the effects of match-related factors, respectively. Results showed higher running performance in the B group compared to the A group during matches. Increased ACC number was seen in matches played on artificial turf; winning was associated with decreased HR_max and increased DEC number. Athletes covered higher TD reaching lower HR_max when playing against lower-ranking opponents while matches lost/drawn resulted in higher HSR. High HRs (max and avg) were found when playing against a team with a positive performance trend. In summary, match-related factors and opponents’ characteristics can affect athletic performance in female soccer athletes. These factors should be considered during in-seasonal training to personalize match preparation and optimize athlete performance.

## 1. Introduction

Over the last decade, interest and investment in female sports have grown significantly, leading to noteworthy improvements in performance, participation, professionalism, and scientific research [1]. Particularly, female soccer has emerged as a prominent area for investment, increasing participation and enhancing performance and research through new technologies [2]. Indeed, nowadays, professional teams largely use different technologies to monitor external and internal load to increase performance while decreasing injury risks. One of the most used technologies is the Global Positioning System (GPS) integrated with accelerometry, gyroscope sensors, and heart rate (HR) monitors [3,4,5]. These instruments allow the monitoring of different running and HR variables, such as total running distance (TD), distance covered at high speed (HSR), distance covered while sprinting (SPR), acceleration number (Acc_n) and deceleration number (Dec_n), and HR features [3,4,6,7,8,9,10,11].

A scoping review conducted by Pérez et al. [1] provides average performance data for female soccer, highlighting that TD during match play is about 9556 ± 795 m and, considering intensity zones, that athletes performed about 830 ± 1414 m and 267 ± 275 m of HSR and SPR, respectively.

The variability in the number of accelerations and decelerations across studies, influenced by methodological differences, underscores the huge challenge when interpreting these findings [12]. This variability is particularly pronounced across different positional units and starting speeds; however, understanding the distances covered during accelerations and decelerations is crucial for grasping sport and level-specific demands [13].

Moreover, while absolute movement metrics are crucial for describing sport characteristics and individual external load, per-minute relative metrics are essential for assessing an athlete’s pace and condition independently of total playing time [14]. This approach facilitates comparisons between athletes with varying playing durations, controlling biases from shorter playing times. By normalizing data, per-minute metrics ensure a fair evaluation of performance intensity and efficiency, allowing coaches to make more informed decisions about player fitness and readiness and to evaluate athletes who played the whole game alongside those who played only a part of it.

Research in male soccer has traditionally been more extensive than in female soccer, focusing on various influencing factors; in contrast, comparable transparency and depth of analysis are often lacking in studies on female soccer. Articles on male soccer have identified several contextual factors related to both the environment and match dynamics that influence athletes’ performance. For example, the playing surface significantly affects running performance variables in football, as suggested by Vescovi et al. (2019) [11], who provided data supporting the greater efficacy of artificial turf compared to natural grass; however, they presented contrasting results based on subjective measures [14]. Regarding match-related factors, teams exhibit different performance metrics based on match outcomes and opponent strength: winning teams tend to demonstrate greater TD, increased SPR, and a higher number of Acc, whereas drawing or losing teams tend to show higher values in HSR metrics [6]. Playing against higher-ranked teams often results in increased TD, HSR, and Acc, with higher SPR distances in losses and lower HSR distances in wins [4,6]. Conversely, when facing lower-ranked teams, SPR values tend to be higher, with decreased TD and Acc observed in losses [4,6]. Furthermore, winning teams show less high-intensity activity during matches, suggesting a strategy of energy conservation with a slower pace, whereas teams in losing or drawing positions exert fewer low-intensity efforts to recover [15]. Female soccer players have different physiological, biomechanical, and psychological characteristics compared to male soccer players, and, considering the limited literature on this topic in the female gender, it is necessary to produce evidence on women’s soccer. Furthermore, knowing how some contextual factors—such as the field surface, tactical aspects, and the level of the opponents—could influence performance differently compared to men’s soccer could play a key role in creating specific and more effective training protocols for female soccer players.

Although several studies offer a team overview of the external load, the distinction between playing roles is sometimes overlooked [3,7,8,15], while others classify athletes according to their role [4,5,6,9,11,12]. Notably, significant differences in running parameters have been observed between attackers, midfielders, and defenders, highlighting variations in athletic and physical capabilities both within and between roles [9].

Therefore, this study aims to evaluate the impact of match-related variables, such as playing surface, opponent levels, opponent trends, match status, and match outcomes, on the athletic performance of professional female soccer players throughout an entire competitive season in the Italian second division. To our knowledge, the study of opponent trends (outcomes of the last three matches) and match status (matches played in winning or losing/drawing situations) represents a new area of research never analyzed before in women’s soccer, although such trends’ impact on performance could be very influential. Hence, understanding the influence of these factors on players’ performance during matches could provide new insights and help in building match-specific training schedules during the season. Moreover, analyzing the differential effect of match-related factors on distinct playing roles could guide technical staff in their choice of match players. Finally, analyzing data minute by minute could produce more comprehensive information compared to the existing literature and could increase the applicability of results in training scenarios.

The authors of this study hypothesized that athletes who showed different athletic performances in fitness tests and belonged to different playing positions would show significant differences in athletic performances during matches. This study also hypothesized that match-related factors, such as playing surface, opponent levels, opponent trends, match status, and match outcomes, significantly influence the soccer performance of professional female athletes and could have a different impact on players with different playing roles.

## 2. Materials and Methods

### 2.1. Partecipants

Study participants played in the first team of S.S. Lazio Women, competing in the second-highest division of the Italian soccer championships. To be included in this study, players were required to fulfil the following criteria:

- Being healthy and unaffected by any physical or psychological issues that could have affected the performance during the season.
- Having played at least 60 min of each analyzed match.

Although the total roster of the team consisted of twenty-five elite athletes, only a limited number of players met the inclusion criteria and competed in all championship matches. Thus, seventeen professional female soccer players (mean age 25.5 ± 4.3 years; body mass index 21.6 ± 2.0 kg/m^2^) participated in this study.

To analyze athletes’ performance in relation to specific players’ characteristics, all athletes were divided into two groups according to their roles during matches.

- Group A: central defenders and forwards (eleven players).
- Group B: right and left full-backs and midfielders (six players).

The game system and the tactical strategy determined the allocation of player roles into the two groups. The team played in a 4-3-1-2 formation throughout the regular season. In this formation, the development of both offensive and defensive playing actions leads the midfielders and full-backs to travel long distances compared to the attackers and central defenders. At the beginning of the defensive actions, the team presses the opposing full-backs high with the external midfielders, while in the offensive actions, it uses continuous overlaps between the full-backs and the external midfielders. In both of these game phases, the central midfielders make continuous movements to cover or support the action. These elements allowed us to distinguish the work carried out by these roles compared to that of forwards and central defenders. Furthermore, the literature describes running data for the different roles regardless of the game module, and these data show values that well support our distribution criterion [16,17].

### 2.2. Ethical Approval

All athletes were informed about the project through a detailed written document. Informed consents were collected before the study began. The institutional ethics committee of Niccolò Cusano University (protocol number MO3-22) granted ethical approval in accordance with the 1964 Helsinki Declaration and its subsequent amendments or comparable ethical standards.

### 2.3. Study Protocol

This study was performed by analyzing 22 matches of the 2022/2023 competitive season (from September 2022 to May 2023). At the end of the championship, the team ranked second in the league, playing an entire season at the top of the tournament. Considering that athletes with similar roles can have different physical and fitness characteristics, to avoid methodological bias, at the end of the pre-season period, all athletes underwent two sessions of physical fitness tests. For the duration of the whole competitive season, match-related factors were collected. In addition, the athletes’ internal and external workload was recorded for each match.

Physical fitness measurements:

Before the beginning of the championships (August 2022), athletes underwent two sessions of physical tests. During day one, athletes performed squat jumps (SJs), countermovement squat jumps (CMJs) with blocked arms, and monopodalic CMJs (mCMJs) for the right and left leg tests. These tests were performed using the Optojump System (96 leds, 1.0416 cm resolution, Microgate, Bolzano, Italy), and the height of each jump was recorded. Additionally, athletes performed a 20 m linear sprint test (LS) and a 20 m sprint test, with right and left changes of direction (CodS) tested separately (Cod at 90° after 10 m). The time in sprint tests was detected by two pairs of dual-beam photocells (Witty, Microgate, Bolzano, Italy). For each test, each athlete performed a total of three attempts, and the best score out of three was used for data analysis.

On the second testing day, athletes performed the yo-yo intermittent recovery test level 1 (Yo-Yo IR1) to assess the players’ aerobic capability. The measure of the total covered distance was calculated and used for further analysis [18].

Internal and external workload measurements:

To collect workload data, players were monitored using GPS devices (GPEXE, recording frequency 20 Hz, Exelio srl, Udine, Italy) during the analyzed matches. Total distance (TD), sprint distance (>22.5 km/h; SD), high-speed running (>15 km/h; HSR), acceleration (>2 m/s^2^), and deceleration (<2 m/s^2^) numbers (Acc_n; Dec_n) were used to calculate match volume and intensity. To obtain comparable data for each player, the recorded GPS variables were classified by actual game time (total playing minutes). Therefore, it was possible to analyze comparable data and avoid bias due to the analysis of just athletes who were able to play the whole game.

In addition, maximal (HR_Max) and average (HR_Avg) heart rates were recorded by a wearable heart rate sensor (Polar H9) to measure athletes’ cardiovascular effort during matches.

Match-related factor collection:

The following match-related factors were collected during the matches in order to create a database of the season:

- Type of surface (natural or artificial grass);
- Match outcome (win, draw, or loss);
- Opponent level (highest: 1st to 5th, medium: 6th to 10th, and lowest: 11th to 16th placement at the end of the championship);
- Match status (matches played in winning or losing/drawing situations for more than half of the playing time);
- Opponent positive or negative trends (outcomes of the last three matches; a weighted score average with greater weight assigned to most recent matches).

### 2.4. Statistical Analysis

Data are reported as mean ± standard deviation. To check normality distribution and homogeneity of variance, the Shapiro–Wilk test and Levene’s test were performed. Then, a natural log transformation was applied to two of the seven variables that were not normally distributed (Dec_n and HR_Avg). An unpaired *t*-test was performed to detect differences in mean values in fitness tests between Group A and Group B. To estimate the differential effects of each analyzed factor on Group A and Group B, a mixed-model analysis of variance (2 × 2 and 2 × 3 ANOVA) was used. The analyses of the main effects of match-related factor conditions (carried out on two levels for type of surface; match outcome; match status; and opponent trends and on three levels for opponent level), groups (on two levels for Group A vs. Group B), and the interaction between them (condition × group) were then performed. When significant main effects or interactions were found, a Bonferroni post hoc analysis was used to interpret data. The statistical significance level was set at *p* ≤ 0.05.

## 3. Results

### 3.1. Physical Fitness Test

The statistical analysis revealed a significant difference between groups in seven out of eight tests of the fitness battery. In particular, Group B showed better scores compared to Group A in the jumping tests (SJ, CMJ, mCMJ-l) in the sprint tests (LS, Cod-r, Cod-l), and in the Yo-Yo IR1 test. The mean and standard deviation values of each test are shown in Table 1.

### 3.2. Effect of Groups in GPS and HR Variables

The ANOVA revealed that significant differences (*p* < 0.01) between groups were found in all measured GPS variables except for acceleration and deceleration numbers. In particular, Group B values of TD, SD, HSR, HR_Max, and HR_Avg were higher than values of Group A. The mean and standard deviation of all GPS variables recorded in the 22 matches analyzed for both groups are shown in Table 2.

### 3.3. Effects of Match-Related Factors on GPS and HR Variables

ANOVA revealed that each match-related factor had a significant effect on at least one of the measured variables. Overall, the analyzed factors showed significant effects on TD, HSR, Acc_n, Dec_n, and HR variables. All ANOVA results of the main effect of condition and condition × group interaction are reported in Table 3.

**Figure 1:**
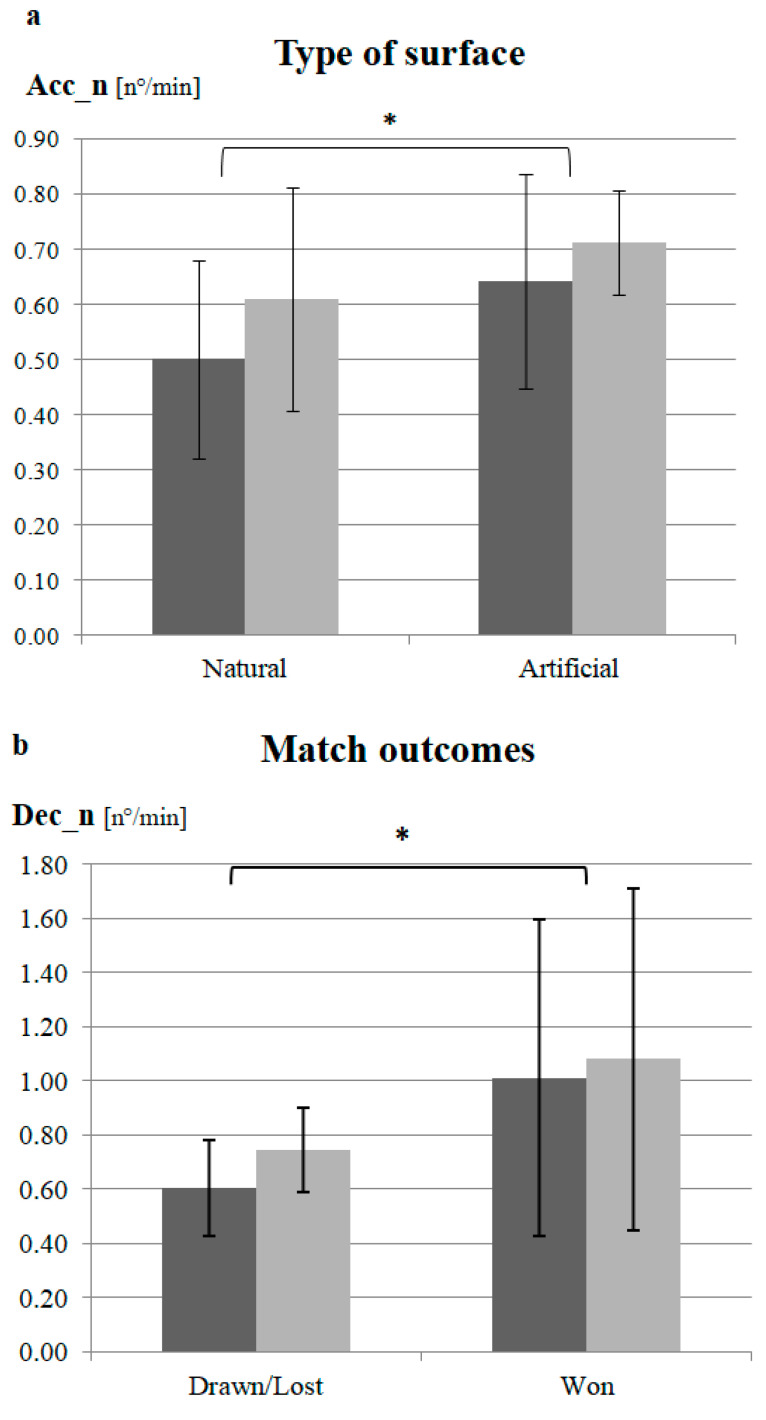

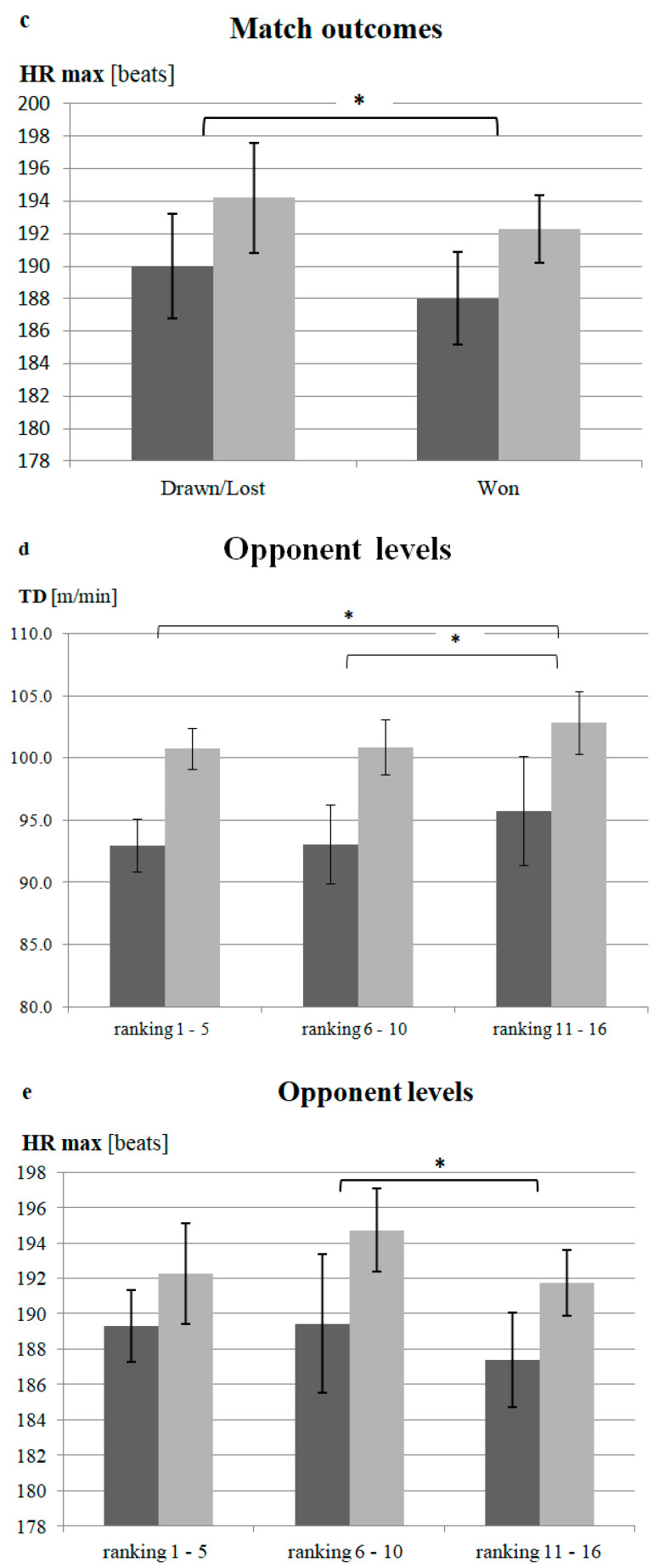

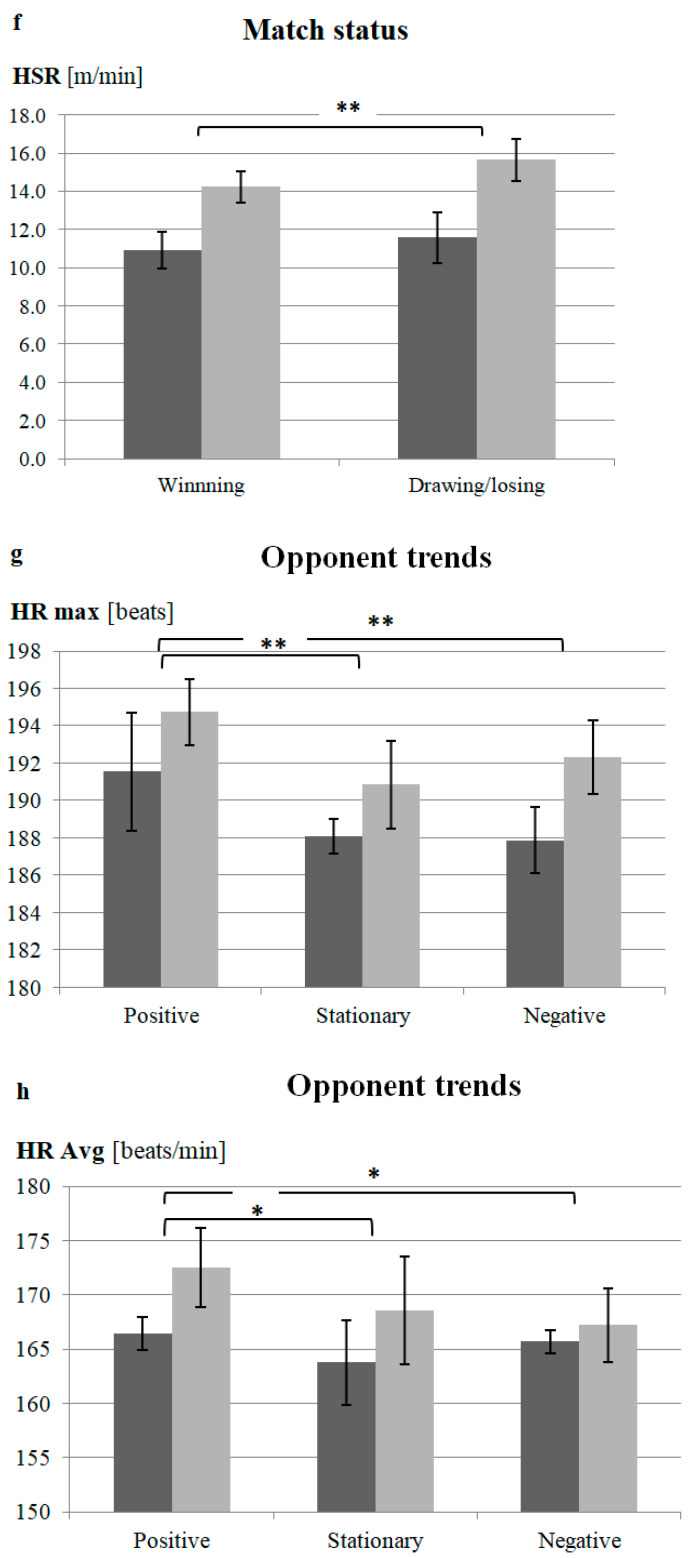
(**a**–**h**) Dark gray: Group A; light gray: group B. (**a**) Acceleration numbers and type of surface; (**b**) deceleration numbers and match outcomes; (**c**) Maximal heart rate and match outcomes; (**d**) Total distance covered and levels of opponent; (**e**) Maximal heart rate and levels of opponent; (**f**) High speed running and match status; (**g**) Maximal heart rate and trends of opponent; (**h**) Average heart rate and trends of opponent. Significant differences by match-related conditions: * *p* ≤ 0.05; ** *p* < 0.01.

Concerning the type of surface, a significant effect of condition was found. In particular, when athletes played on artificial grass, they had a significantly higher Acc_n compared to matches on natural grass (0.68 ± 0.16 n°/min vs. 0.56 ± 0.19 n°/min, respectively; *p* = 0.05) (Figure 1a). No significant condition or condition × group effects were found in the other GPS variables for the type of surface.

A significant effect of the final match score was found in two GPS parameters. Specifically, both higher Dec_n (won = 1.05 ± 0.60 n°/min; drawn/lost = 0.67 ± 0.17 n°/min; *p* = 0.047) and significantly lower HR_Max (won = 190.16 ± 3.28 bpm; drawn/lost = 192.10 ± 3.86 bpm; *p* = 0.042) were observed in matches won compared to matches lost or drawn (Figure 1b,c). Concerning opponent level, a significant effect of condition was found in TD and HR_Max. In particular, when athletes played against lower-ranked teams, they covered higher TD (high-ranked = 96.87 ± 4.45 m/min vs. low-ranked = 99.28 ± 5.03 m/min; *p* = 0.034; middle-ranked = 96.96 ± 4.83 m/min vs. low-ranked; *p* = 0.032) reaching a lower HR_Max (middle-ranked = 192.07 ± 4.14 bpm vs. low-ranked = 189.55 ± 3.16 bpm; *p* = 0.012) compared to the TD and HR_Max of higher-ranked opponents, respectively (Figure 1d,e). In matches played with more than half of the time losing/drawing, players showed significantly higher HSR (winning = 12.57 ± 1.91 m/min vs. losing/drawing = 13.61± 2.39 m/min; *p* = 0.002) compared to matches played winning (Figure 1f). Analyzing the performance trends of the opponents, the results showed that significantly higher levels of HR_Max (positive = 193.14 ± 2.95 bpm vs. stationary = 189.47 ± 2.22 bpm; *p* < 0.001; positive vs. negative = 190.09 ± 2.93 bpm; *p* = 0.001) and HR_Avg (positive = 169.47 ± 4.17 bpm vs. stationary = 166.16 ± 4.94 bpm; *p* = 0.019; positive vs. negative = 166.45 ± 2.57 bpm; *p* = 0.022) were reached when playing against opponents with positive trends (i.e., a high score in the last three matches) compared to opponents with stationary or negative trends (Figure 1g,h). No significant condition or condition × group effects were found in the other GPS or HR variables and match-related factors.

## 4. Discussion

This study is the first to examine how match-related factors, such as match status and opponent trends, affect the running performance and HR variables of elite female soccer players. Moreover, this study clustered athletes by roles that have also shown significant differences in fitness level (central defenders and forwards; right and left full-backs and midfielders).

Previous research has focused on specific roles on the field, such as defenders, midfielders, and attackers [9], but none have categorized players by physical fitness level determined through physical tests. Athletes playing the same role could indeed show different values of athletic performance due to their individual morphological and physiological characteristics. To verify the correct role clustering, this study first analyzed significant differences in fitness levels between the two groups (found in seven out of eight fitness tests). The results showed that these differences were consistent with the running and cardiovascular performance observed with GPS and HR monitoring during matches, except for Acc_n and Dec_n. This exception may be due to the methodology of considering acceleration and deceleration within the broader context of soccer performance, where speed also plays a crucial role [12]. Further research could delve into this sub-topic, considering the growing importance of explosive strength in modern soccer strategies and training. The existing literature [9] has indicated that different roles require varying physical demands, as attackers and midfielders perform more frequent and intense running efforts compared to defenders [19,20].

Additionally, this study used a per-minute based analysis of GPS and HR data, which enabled comparisons between athletes who played for different amounts of time in each match. In fact, some previous studies [11] used convenient samples (athletes who played at least 90 min per match) to obtain comparable data. However, using this selection criterion could lead to sample bias; only athletes with high athletic performance and/or starters, would be included in this analysis. The inclusion criterion of this study (having played at least 60 min of each analyzed match) in addition to the per-minute analysis provided a more realistic overview of the average athletes’ performance, limiting sample selection bias.

Regarding match-related factors, the results showed a significant main effect of condition in some variables, but no condition × group difference was observed in any analyzed factor. Hence, even if Groups A and B showed significantly different running and cardiovascular performance while playing, match-related factors demonstrated a comparable influence in both groups. Therefore, the results of this study did not confirm our hypothesis about the differential effects of match-related factors on the two groups of athletes. In other words, these findings suggest that the analyzed factors could impact athletic performance during matches, independently of athletic players’ characteristics and their roles.

Regarding type of surface, the results showed that the team recorded significantly more accelerations when competing on artificial turf compared to natural grass, suggesting that high-intensity movements are more efficient on artificial turf. Two plausible explanations are supported by the literature: first, the interaction between players’ shoes and the turf surface, along with enhanced grip, likely facilitated an increase in players’ speed potential; second, the increased rapidity of ball movement on artificial turf may require players to elevate their movement speed accordingly [11]. Indeed, Roberts and colleagues (2020) qualitatively confirmed that artificial turf makes ball–surface interactions more predictable due to the uniformity of the grass, allowing players to increase high-intensity efforts [14]. Additionally, the growing professionalism in women’s football and new approaches in the design and realization of third-generation (3G) artificial and ecological turf may further enhance performance [19,21,22]. This type of turf is more resistant than natural grass, allowing for higher usage with less decay and longer duration. It is important to note that the artificial turf considered in most of the matches was of high quality, contributing to the observed performance improvements. However, the evaluation of the surface depends also on other performance-related factors; indeed, a qualitative study has indicated that athletes prefer natural turf to artificial turf due to perceived lower injury risk and greater shock absorption coming from the natural field [23].

The opponent level and the last matches’ trend played a significant role in running and HR variable changes. When playing against weaker teams or those on a losing streak, players exhibited lower effort and running performance. The data show a reduction in HR_Max and an increase in the number of decelerations when the team won. This is consistent with results on men’s professional soccer obtained by Castellano et al. [24], who found that players tend to exert more effort against evenly matched opponents, driven by an amplified psychological motivation to secure victory. While increased decelerations are not always linked to winning outcomes [25], they may indicate a greater need to defend positions, thereby reducing goal opportunities for opponents. Additionally, decelerations and accelerations are associated with game intensity [26], reflecting high-intensity movement changes. Another explanation could be that when playing against weaker teams, players can more easily change the pace of the game and run faster, requiring more frequent decelerations. Additionally, when competing against lower-ranked teams with significant skill gaps, the exerted effort diminishes even as the TD covered increases, with GPS data confirming a decrease in HR_Max and an increase in TD covered in these situations. According to Curtis et al. [5], male soccer matches against lower-ranked opponents tend to feature slower ball movements, leading to reduced internal and external exertion and a decreased pace in the entire game. Conversely, facing challenging opponents results in an increased effort, driven by the team’s psychological desire to win. The results of this study are further supported by higher internal loads, both maximal and average, when playing against opponents with a positive performance trend, indicating intensified effort and determination to win. Similarly, when the team experienced a match with a situation of loss or draw, there was an increase in HSR activity, reflecting a greater effort to overturn match results. This trend is in line with the results of the study by Aquino and colleagues [6], who demonstrated higher demands for high-intensity running when drawing compared to winning. The team of this study was from the top of the championship table, leading to a consistent desire to win every match and the confidence to do so. Thus, when the team was losing or drawing, the psychological drive was to increase the effort to recover and secure the victory. Overall, the results of this study could provide additional information to the technical staff and athletes themselves, in order to guide training choices during the season. For example, within the championship, carrying out specific drills when playing on different field surfaces would be advisable to allow the body to adapt better and prevent injuries. Moreover, it would be possible to adjust training schedules and tactical strategies based on the contextual factors of upcoming matches, particularly concerning psychological factors and tactical aspects, according to the study of the opponents’ playing systems and the strategies they adopt. Finally, it is possible to work on the mental aspect of athletes to provide the right stimuli when facing lower-level teams and to implement specific training to manage the match’s partial result during advantageous or disadvantageous situations.

## 5. Conclusions

In conclusion, this study underscores the importance of considering not only playing positions but also physical fitness level when tailoring training programs. By accounting for match-related factors, it is possible to better understand the demands of each role in various situations. This study suggests that opponent level and trends influence the running and cardiovascular soccer performance of female athletes. In light of these findings, match-related factors and opponent characteristics should be considered during in-season training, since they can affect athletic performance in female soccer athletes. The analysis of these parameters could help in individualizing match preparation and optimizing athletes’ performance, allowing coaches and trainers to develop appropriate strategies related to a specific match.

The limitations of this study include its small sample size, which likely lowered the statistical power of the analyses performed. However, the choice of a limited number of athletes from the same professional team competing in for the whole championship season enabled us to exclude other confounding factors that could have influenced the study results. Another limitation is the lack of differentiation between ball possession and non-possession phases, which could have led to additional insights. Future research should incorporate this distinction, as it is crucial for understanding differences between offensive and defensive phases, justifying variables such as acceleration and deceleration, and providing a clearer overall picture.

## Figures and Tables

**Table 1:** Group mean and standard deviation results of fitness tests.

Jump Test	SJ [cm]	CMJ [cm]	mCMJ-r [cm]	mCMJ-l [cm]
**Group A**	**25.59 ± 3.57**	**26.88 ± 3.88**	18.01 ± 2.60	**18.05 ± 2.98**
**Group B**	29.68 ± 1.84 *	30.44 ± 1.55 *	19.87 ± 1.10	20.88 ± 1.57 *
**Sprint and Yo-Yo IR1 test**	**LS [s]**	**CodS-r [s]**	**CodS-l [s]**	**Yo-Yo IR1 [m]**
**Group A**	**3.67 ± 0.31**	**4.26 ± 0.10**	**4.27 ± 0.11**	**1076.36 ± 275.94**
**Group B**	3.35 ± 0.10 *	4.15 ± 0.10 *	4.12 ± 0.11 *	1396.67 ± 267.26 *

Table legend: SJ: squat jump; CMJ: countermovement jump; mCMJ-r: monopodalic jump with right leg; mCMJ-l: monopodalic jump with left leg; LS: linear sprint; CodS-r: sprint with the right change of direction; CodS-l: sprint with the left change of direction; Yo-Yo IR1: Yo-Yo intermittent recovery test level 1. * *p* < 0.05; Group A vs. Group B.

**Table 2:** Group mean and standard deviation results of GPS variables.

GPS Variables	Group A	Group B	*p*	*95% Conf. Int. of the Difference*
**TD [m/min]**	94.13 ± 3.62	101.64 ± 2.33	0.000 *	−9.35; −5.65
**SD [m/min]**	0.96 ± 0.24	1.19 ± 0.23	0.002 *	−0.37; −0.08
**Acc_n [n°/min]**	0.54 ± 0.19	0.64 ± 0.18	0.084	−0.21; 0.01
**Dec_n [n°/min]**	0.90 ± 0.53	0.99 ± 0.56	0.598	−0.42; 0.24
**HSR [m/min]**	11.24 ± 1.17	14.93 ± 1.19	0.000 *	−4.41; −2.98
**HR_Max [beats]**	188.56 ± 3.02	192.83 ± 2.57	0.000 *	−5.97; −2.56
**HR_Avg [beats/min]**	165.04 ± 3.15	168.98 ± 4.31	0.001 *	−6.23; −1.63

Legend: TD: total distance; SD: sprint distance; Acc_n: acceleration number; Dec_n: deceleration number; HSR: high-sprint running; HR_Max: maximal heart rate; HR_Avg: heart rate average. * *p* < 0.01 Group A vs. Group B.

**Table 3:** ANOVA of match-related factor conditions and condition × group interaction. * *p* < 0.05 Group A vs. Group B. Effect size (ƞ^2^) thresholds: small effect <0.06, moderate effect between >0.06, and <0.14, and large effect >0.14.

	Factor	Variable	F	*p*	ƞ^2^	Observed Power	Variable	F	*p*	ƞ^2^	Observed Power	df	Error
**Type of surface**	Condition	**TD**	0.15	0.70	0.00	0.07	**SD**	0.55	0.46	0.01	0.11	1	40
	Condition × Group		0.52	0.47	0.01	0.11		0.01	0.93	0.00	0.05		
	Condition	**Acc_n**	3.99	0.05 *	0.09	0.50	**Dec_n**	1.50	0.23	0.04	0.22		
	Condition × Group		0.10	0.76	0.00	0.06		0.08	0.78	0.00	0.06		
	Condition	**HSR**	0.21	0.65	0.01	0.07	**HR_Max**	0.25	0.62	0.01	0.08		
	Condition × Group		0.20	0.66	0.00	0.07		0.06	0.81	0.00	0.06		
	Condition	**HR_Avg**	0.19	0.67	0.00	0.07							
	Condition × Group		0.05	0.83	0.00	0.06							
**Match outcome**	Condition	**TD**	0.01	0.92	0.00	0.05	**SD**	0.33	0.57	0.01	0.09	1	40
	Condition × Group		0.31	0.58	0.01	0.08		0.28	0.60	0.01	0.08		
	Condition	**Acc_n**	0.25	0.62	0.01	0.08	**Dec_n**	4.22	0.047 *	0.10	0.52		
	Condition × Group		0.01	0.91	0.00	0.05		0.04	0.84	0.00	0.05		
	Condition	**HSR**	3.05	0.09	0.07	0.40	**HR_Max**	4.42	0.042 *	0.10	0.54		
	Condition × Group		0.00	0.98	0.00	0.05		0.00	0.96	0.00	0.05		
	Condition	**HR_Avg**	0.68	0.42	0.02	0.13							
	Condition × Group		0.95	0.34	0.02	0.16							
**Opponent level**	Condition	**TD**	3.45	0.042 *	0.15	0.61	**SD**	0.76	0.48	0.04	0.17	2	38
	Condition × Group		0.08	0.93	0.00	0.06		0.49	0.62	0.03	0.12		
	Condition	**Acc_n**	0.81	0.45	0.04	0.18	**Dec_n**	2.36	0.11	0.11	0.45		
	Condition × Group		0.10	0.91	0.01	0.06		0.08	0.92	0.00	0.06		
	Condition	**HSR**	0.50	0.61	0.03	0.13	**HR_Max**	3.52	0.039 *	0.16	0.62		
	Condition × Group		2.30	0.11	0.11	0.44		0.60	0.55	0.03	0.14		
	Condition	**HR_Avg**	1.37	0.27	0.07	0.28							
	Condition × Group		1.66	0.20	0.08	0.33							
**Match situation**	Condition	**TD**	0.03	0.86	0.00	0.05	**SD**	2.29	0.14	0.05	0.31	1	40
	Condition × Group		0.32	0.57	0.01	0.09		1.86	0.18	0.04	0.26		
	Condition	**Acc_n**	1.86	0.18	0.04	0.27	**Dec_n**	3.78	0.06	0.09	0.48		
	Condition × Group		0.24	0.63	0.01	0.08		0.01	0.92	0.00	0.05		
	Condition	**HSR**	10.65	0.002 *	0.21	0.89	**HR_Max**	1.77	0.19	0.04	0.25		
	Condition × Group		1.37	0.25	0.03	0.21		0.00	0.99	0.00	0.05		
	Condition	**HR_Avg**	0.03	0.87	0.00	0.05							
	Condition × Group		0.06	0.80	0.00	0.06							
**Formation tactics**	Condition	**TD**	3.13	0.08	0.07	0.41	**SD**	0.08	0.77	0.00	0.06	1	40
	Condition × Group		0.48	0.49	0.01	0.10		0.14	0.71	0.00	0.07		
	Condition	**Acc_n**	1.43	0.24	0.03	0.21	**Dec_n**	4.69	0.036 *	0.10	0.56		
	Condition × Group		0.25	0.62	0.01	0.08		0.33	0.57	0.01	0.09		
	Condition	**HSR**	0.21	0.65	0.01	0.07	**HR_Max**	0.00	0.98	0.00	0.05		
	Condition × Group		0.65	0.42	0.02	0.12		0.66	0.42	0.02	0.12		
	Condition	**HR_Avg**	0.01	0.94	0.00	0.05							
	Condition × Group		1.39	0.25	0.03	0.21							
**Opponent trend**	Condition	**TD**	2.11	0.14	0.11	0.40	**SD**	0.73	0.49	0.04	0.16	2	34
	Condition × Group		0.43	0.65	0.02	0.11		1.02	0.37	0.06	0.21		
	Condition	**Acc_n**	1.23	0.30	0.07	0.25	**Dec_n**	2.17	0.13	0.11	0.41		
	Condition × Group		0.04	0.96	0.00	0.06		0.02	0.98	0.00	0.05		
	Condition	**HSR**	2.08	0.14	0.11	0.40	**HR_Max**	11.03	0.0002 *	0.39	0.99		
	Condition × Group		0.20	0.82	0.01	0.08		0.63	0.54	0.04	0.15		
	Condition	**HR_Avg**	3.86	0.031 *	0.19	0.66							
	Condition × Group		1.85	0.17	0.10	0.36							

## Data Availability

Data are available upon request to the contact author.

## References

[1] Pérez Armendáriz M.L., Spyrou K., Alcaraz P.E. (2024). Match demands of female team sports: A scoping review. Biol. Sport.

[2] Fédération Internationale de Football Association (FIFA) (2018). Women’s Football Strategy.

[3] González-García J., Giráldez-Costas V., Ramirez-Campillo R., Drust B., Romero-Moraleda B. (2023). Assessment of peak physical demands in elite women soccer players: Can contextual variables play a role?. Res. Q. Exerc. Sport.

[4] Trewin J., Meylan C., Varley M.C., Cronin J., Ling D. (2018). Effect of match factors on the running performance of elite female soccer players. J. Strength Cond. Res..

[5] Curtis R.M., Huggins R.A., Benjamin C.L., Sekiguchi Y., Adams W.M., Arent S.M., Jain R., Miller S.J., Walker A.J., Casa D.J. (2020). Contextual factors influencing external and internal training loads in collegiate men’s soccer. J. Strength Cond. Res..

[6] Aquino R., Guimarães R., Junior GO C., Clemente F.M., García-Calvo T., Pulido J.J., Nobari H., Praça G.M. (2022). Effects of match contextual factors on internal and external load in elite Brazilian professional soccer players through the season. Sci. Rep..

[7] Augusto D., Brito J., Aquino R., Figueiredo P., Eiras F., Tannure M., Veiga B., Vasconcellos F. (2021). Contextual variables affect running performance in professional soccer players: A brief report. Front. Sports Act. Living.

[8] García-Unanue J., Pérez-Gómez J., Giménez J.V., Felipe J.L., Gómez-Pomares S., Gallardo L., Sánchez-Sánchez J. (2018). Influence of contextual variables and the pressure to keep category on physical match performance in soccer players. PLoS ONE.

[9] Griffin J., Larsen B., Horan S., Keogh J., Dodd K., Andreatta M., Minahan C. (2020). Women’s football: An examination of factors that influence movement patterns. J. Strength Cond. Res..

[10] Owen A.L., Wong del P., McKenna M., Dellal A. (2011). Heart rate responses and technical comparison between small- vs. large-sided games in elite professional soccer. J. Strength Cond. Res..

[11] Vescovi J.D., Falenchuk O. (2019). Contextual factors on physical demands in professional women’s soccer: Female Athletes in Motion study. Eur. J. Sport Sci..

[12] Mara J.K., Thompson K.G., Pumpa K.L., Morgan S. (2017). The acceleration and deceleration profiles of elite female soccer players during competitive matches. J. Sci. Med. Sport.

[13] Torres-Ronda L., Beanland E., Whitehead S., Sweeting A., Clubb J. (2022). Tracking Systems in Team Sports: A Narrative Review of Applications of the Data and Sport Specific Analysis. Sports Med. Open..

[14] Roberts J.R., Osei-Owusu P., Mears A.C., Harland A.R. (2020). Elite Players’ Perceptions of Football Playing Surfaces: A Qualitative Study. Res. Q. Exerc. Sport.

[15] Lago C., Casais L., Dominguez E., Sampaio J. (2010). The effects of situational variables on distance covered at various speeds in elite soccer. Eur. J. Sport Sci..

[16] Di Salvo V., Baron R., Tschan H., Calderon Montero F.J., Bachl N., Pigozzi F. (2007). Performance characteristics according to playing position in elite soccer. Int. J. Sports Med..

[17] Rampinini E., Coutts A.J., Castagna C., Sassi R., Impellizzeri F.M. (2007). Variation in top level soccer match performance. Int. J. Sports Med..

[18] Bangsbo J., Iaia F.M., Krustrup P. (2008). The Yo-Yo intermittent recovery test: A useful tool for evaluation of physical performance in intermittent sports. Sports Med..

[19] Andersson H.A., Randers M.B., Heiner-Møller A., Krustrup P., Mohr M. (2010). Elite female soccer players perform more high-intensity running when playing in international games compared with domestic league games. J. Strength Cond. Res..

[20] Hewitt A., Norton K., Lyons K. (2014). Movement profiles of elite women soccer players during international matches and the effect of opposition’s team ranking. J. Sports Sci..

[21] (2021). FIFA Quality Programme for Football Turf. https://inside.fifa.com/technical/football-technology/standards/football-turf/fifa-quality-programme-for-football-turf.

[22] FIFA (2015). FIFA Quality Programme for Football Turf: Handbook of Requirements.

[23] Geertsema C., Geertsema L., Farooq A., Harøy J., Oester C., Weber A., Bahr R. (2021). Injury prevention knowledge, beliefs and strategies in elite female footballers at the FIFA Women’s World Cup France 2019. Br. J. Sports Med..

[24] Castellano J., Blanco-Villaseñor A., Alvarez D. (2011). Contextual variables and time-motion analysis in soccer. Int. J. Sports Med..

[25] Díez A., Lozano D., Arjol-Serrano J.L., Mainer-Pardos E., Castillo D., Torrontegui-Duarte M., Nobari H., Jaén-Carrillo D., Lampre M. (2021). Influence of contextual factors on physical demands and technical-tactical actions regarding playing position in professional soccer players. BMC Sports Sci. Med. Rehabil..

[26] Barrera J., Sarmento H., Clemente F.M., Field A., Figueiredo A.J. (2021). The Effect of Contextual Variables on Match Performance across Different Playing Positions in Professional Portuguese Soccer Players. Int. J. Environ. Res. Public Health.

